# Telerehabilitation Is a Valid Option for Total Knee Arthroplasty Patients: A Retrospective Pilot Study Based on Our Experience during the COVID-19 Pandemic

**DOI:** 10.3390/healthcare11182489

**Published:** 2023-09-07

**Authors:** Michele Venosa, Emilio Romanini, Enrico Ciminello, Simone Cerciello, Massimo Angelozzi, Vittorio Calvisi

**Affiliations:** 1 Department of Life, Health and Environmental Sciences, University of L’Aquila, Via Vetoio, Coppito 2, 67100 L’Aquila, Italy; massimo.angelozzi@univaq.it (M.A.); vittorio.calvisi@univaq.it (V.C.); 2 RomaPro, Polo Sanitario San Feliciano, Via Mattia Battistini 44, 00167 Rome, Italy; emilio.romanini@gmail.com; 3 GLOBE, Italian Working Group on Evidence-Based Orthopaedics, Via Nicola Martelli 3, 00197 Rome, Italy; 4 Italian Implantable Prostheses Registry (RIPI), Italian National Institute of Health, Viale Regina Elena 299, 00161 Rome, Italy; enrico.ciminello@iss.it; 5 Department of Orthopaedics and Traumatology, Fondazione Policlinico Universitario Agostino Gemelli IRCCS, Largo Agostino Gemelli 8, 00168 Rome, Italy; simone.cerciello@me.com; 6 Orthopaedic Department, Casa di Cura Villa Betania, Via Pio IV 42, 00165 Rome, Italy; 7 UOSD, Department of Mini-Invasive and Computer-Assisting Orthopedic Surgery, San Salvatore Hospital, Via L. Natali 1, 67100 L’Aquila, Italy

**Keywords:** telerehabilitation, in-presence rehabilitation, total knee arthroplasty, outpatient rehabilitation

## Abstract

Introduction: Total knee arthroplasty is an effective operation. Post-surgery rehabilitation, based on early and intensive progressive exercise programs, plays a substantial role and telerehabilitation can be an effective safe option. This retrospective study aimed to compare traditional in-presence rehabilitation and telerehabilitation for total knee arthroplasty, based on our experience during the Italian COVID-19 lockdown. Materials and methods: We retrospectively analyzed 164 patients (94 females and 70 males) enrolled in 2020 within 2 weeks after total knee replacement to perform post-operative outpatient rehabilitation. The clinical results of 82 patients (mean age 66.8 ± 10.2 years) performing telerehabilitation with those obtained from a similar cohort of 82 patients (mean age 65.4 ± 11.8 years) performing traditional in-presence outpatient rehabilitation were compared. Clinical outcomes were examined by comparing the gait speed (Time Up and Go-TUG test), the range of motion, the pain intensity (VAS), the functional status (Oxford Knee Score—OKS and Knee injury and Osteoarthritis Outcome Score—KOOS) and the overall satisfaction (Self-administered patient satisfaction scale) 12 weeks after the beginning of the physiotherapeutic protocol. Results: Telerehabilitation was non-inferior to traditional in-presence rehabilitation in all of the investigated areas and no statistical difference in terms of effectiveness was detected at 12 weeks, as confirmed by the respective patient-reported outcome scores such as TUG test (reduced from 20 ± 2 s to 12 ± 1.5 s for the telerehab cohort and from 18 ± 1.5 s to 13.1 ± 2 s for the in-presence rehabilitation one), pain VAS, OKS (improved from 22 ± 1.3 to 36 ± 2.7 for the telerehab cohort and from 23 ± 2.1 to 35.1 ± 4.2 for the in-presence group), KOOS (improved from 46.2 ± 10.2 to 67.4 ± 3.8 for the telerehabilitation cohort and from 48.4 ± 8.4 to 68.3 ± 6.6 for the other group), and the Self-administered patient satisfaction scale (more than two-thirds of patients globally satisfied with the results of their surgery in both groups). Conclusion: The telerehabilitation program was effective after total knee replacement and yielded clinical outcomes that were not inferior to conventional outpatient protocols.

## 1. Introduction

Osteoarthritis is the most common indication for knee replacement in many industrialized countries. The overall number of these procedures is forecasted to increase further in the next years, due to aging, the extension of lifespan, and the reduction of the age threshold of the population undergoing knee joint replacement [1,2]. Total knee arthroplasty (TKA) is an effective option and its efficacy is well documented. Post-surgery rehabilitation is essential to reduce pain, improve function and activities of daily living, and optimize outcomes [3,4,5]. Post-surgical face-to-face physiotherapy (outpatient or home-based) is generally based on early and intensive progressive exercises, usually lasting 6–8 weeks [6,7,8,9].

Nonetheless, it has been proven that an alternative rehabilitation approach is possible: telerehabilitation, defined by Rosen (1999) [10] as the delivery of medical rehabilitation services at a distance using electronic information and communication technologies, allows both assessment and remote monitoring of patients during physical therapy efficiently and safely [11,12] and is an effective safe option in local rehabilitation settings which is becoming increasingly common [13,14,15].

The COVID-19 pandemic with the risk of direct transmission has been a further incentive [16]. Indeed, in February 2020 Italy was the first country in Europe to face the contagion of COVID-19 and the exceptional spread of COVID-19 led the Italian Prime Minister to declare lockdown status on February 23rd. Accordingly, increasingly strict quarantine measures to limit the movements of the population and ensure social distancing were taken [17,18]. During this period, only essential activities were permitted, allowing people to leave their homes only for clear necessities, such as work, health reasons, or shopping for basic needs [19]. Many activities were interrupted, especially those involving human relationships. Therefore Institutional local health services (Italian National Health Service), urged the partner health centers to activate (when and where possible) telerehabilitation platforms and protocols to ensure adequate rehabilitation programs.

This retrospective pilot study analyzed the outcomes of postoperative rehabilitation in patients undergoing TKA, comparing telerehabilitation (performed as an alternative physiotherapeutic modality due to the COVID-19 pandemic evolution and the Italian national lockdown in 2020) with traditional outpatient in-presence rehabilitation. The hypothesis was that telerehabilitation yielded clinical outcomes which were not inferior to conventional outpatient protocols thus representing a valid alternative to in-person rehabilitation where this is difficult to achieve.

## 2. Materials and Methods

Patients who underwent TKA between 1 March 2020 and 30 June 2020, requiring outpatient rehabilitation and enrolled in a telerehabilitation program, were taken into account. A control arm, composed of a similar cohort of consecutive patients undergoing TKA and who performed traditional outpatient in-presence rehabilitation in the same healthcare context was included in the study.

Eligible individuals were patients aged 50 to 80 years, with a BMI < 35, who had undergone TKA for non-traumatic conditions, without cognitive or blurred vision problems, acute systemic infection, active cancer treatment, stroke within 2 years, rheumatic, neurological, or cardiopulmonary conditions limiting the overall physical function. Patients affected by ipsilateral hip osteoarthritis or those who had previously performed rehabilitation for contralateral knee arthroplasty have been also excluded.

Both cohorts were retrospectively analyzed to compare clinical outcomes at follow-up (FU). A preliminary in-presence examination was performed by the chief medical officer (M.V.) to define a tailored physiotherapeutic program both for telerehabilitation and in-presence groups. Post-operative radiographic imaging was included in the medical chart.

This study was conducted in accordance with the principles of the Declaration of Helsinki and Consent for the use of data was obtained before starting the rehabilitation programs from all patients as part of the standard consent process.

Both telerehabilitation and in-presence rehabilitation programs (45-min for each session; three times a week for 12 weeks) included therapeutic exercises of functional mobility: active exercises (seated and supine), soft tissue massage, patellofemoral joint mobilization, isometric quadriceps, hamstrings, and gluteal strengthening exercises, straight leg raise, transfer training and closed chain exercises (when patients demonstrated good muscle strength and pain control). All patients had received preliminary rehabilitative indications from their orthopedic surgeon during their stay in the hospital (4–10 days) and by the chief medical officer during the preliminary in-presence examination. Furthermore, they have received a written rehabilitative protocol (12-week step-by-step guide)—Appendix A. On this basis, patients were instructed to perform a home exercise program at the end of each treatment. The in-presence rehabilitation was carried out in an outpatient face-to-face modality with the individual relationship between patient and therapist. The telerehabilitation program was delivered using the Cisco WebEx^®^ 39.3 video-conference system, given the need to perform a rehabilitative readaptation in a short time, with a ready-for-use easy tool for elderly patients (not used for technological solutions). In any case, the availability of a caregiver providing technological support was essential and appreciated by all the subjects involved. Video and audio data were transmitted over a high-speed Internet connection to allow real-time two-way interactions. The physiotherapist provided remote clinician oversight and instructions to the patient for the duration of the intervention. A final in-presence examination was performed by the chief medical officer (M.V.) to evaluate clinical outcomes and activities of daily living (ADL) assessment at the end of the rehabilitation program (12 weeks program).

## 3. Outcome Measures

The primary outcome was physical and balance performance and walking autonomy, measuring the time required for standing up from a chair, walking straight for 3 m, turning, walking back to the chair, and sitting down, through the Timed Up-and-Go (TUG) test [20].

Secondary outcomes included resting and movement pain intensity (assessed through a 0–10 cm graphic scale—VAS—administered before and after the physiotherapeutic program), knee range of motion (assessed as the mean value of three hand-goniometer measurements), patient overall satisfaction (assessed through the Self-administered patient satisfaction scale for primary and knee arthroplasty) [21] and knee disability (assessed through the Oxford Knee Score—OKS and the Knee Injury and Osteoarthritis Outcome score—KOOS).

The Self-administered patient satisfaction scale for primary and knee arthroplasty, described by Mahomed et al. in 2011 is a four-item scale focusing on satisfaction in terms of pain relief, increased ability in home or garden activities, ability to do recreational activities, and overall satisfaction with joint mobility. The results are scored on a Likert scale with a total score of 25 to 100 points for each question [21].

The OKS is a patient-reported outcome measure developed in 1998 and approved to assess function and pain after total knee replacement. It consists of 12 questions describing daily life activities and how they have been affected by pain in the previous 4 weeks [22,23]. The KOOS, first developed in 1995 by Ewa M Roos et al. as an extension of the WOMAC Osteoarthritis Index, is a valid, reliable, and sensitive tool to assess function and symptoms in subjects with a knee injury and osteoarthritis. The KOOS is self-administered and takes about 10 min to be filled out. It has five subscales: Pain, other Symptoms, Function in daily living (ADL), Function in Sport and Recreation (Sport/Rec), and knee-related Quality of Life (QOL). Scores are percentages between 0 and 100 (0 = extreme problems; 100 = no problems). The KOOS can be used for short-term and long-term evaluation of several knee pathologies including osteoarthritis [7,24].

## 4. Statistics

Patients’ characteristics and outcome measures were reported on aggregate in terms of mean ± standard deviation for continuous variables and absolute frequencies(percentages) for categorical. Statistical analysis of the outcomes included the significance of differences. The two arms were examined and compared on baseline characteristics and changes in outcomes. The Wilcoxon test for paired samples was used to assess the intra-group difference and the effect of the physiotherapeutic path. A Mann-Whitney *U* test for two independent samples was used to detect significance in differences between groups and assess the inter-group difference and the effect of telerehabilitation. The χ^2^ test was used to check for homogeneity of categorical variables distribution along the two arms. Statistical significance was set at *p* < 0.05 and the Effect Size (ES) for the Mann-Whitney *U* test was computed in the case of the inter-groups test. All statistical analyses were performed using R version 4.2.3 (15 March 2023 ucrt)—“Shortstop Beagle”.

## 5. Results

A total of 164 patients (82 for each group) were included in the study, 48 females and 34 males with 66.8 ± 10.2 years of age on average for the telerehab group and 46 females and 36 males with mean age equal to 65.4 ± 11.8 years for in-presence rehab group. No statistically significative differences were detected in baseline demographics between the two groups, as reported in Table 1

The groups differ in a statistically significant way in all clinical outcome measures but KOOS, for which the difference is nonsignificant. The Telerehab group has better metrics in terms of Range of Motion (Flexion and Extension deficit), while the in-presence group reports a better baseline condition in terms of resting and movement pain (Table 2).

All parameters (pain, function, range of motion, quality of life) improved from baseline to the end of the rehabilitation procedures in both groups. There was no statistical difference between telerehabilitation and traditional in-presence rehabilitation in terms of effectiveness at 12 weeks for some metrics such as movement pain, flexion, and KOOS. On the other hand, a better decrease in pain when resting was observed for patients treated via telerehabilitation, while the best improvement in extension deficit was observed for patients in the in-presence rehabilitation group (*p* < 0.01). TUG test results improved by 8 ± 2.6 s for the telerehabilitation group and by 4.9 ± 2.5 s for the in-presence rehabilitation group (*p* < 0.01). Moreover, improvement in OKS was better for patients treated in telerehabilitation (*p* < 0.01), with the score increasing by 14 ± 3.1 points concerning baseline on average, compared to the improvement of the in-presence group. The inter-group difference for KOOS was not statistically significant, as it improved from 46.2 ± 10.2 to 67.4 ± 3.8 for the telerehabilitation cohort and from 48.4 ± 8.4 to 68.3 ± 6.6 for the other group. The effect size for all test comparisons results to be small (ES < 0.2), but for the TUG test, for which it is moderate (0.2 < ES < 0.5). Self-administered patient satisfaction scale showed no statistically significant differences in any of the considered items: more than two-thirds of patients were globally satisfied with the results of their surgery in both groups. The overall data are available in Table 3, Table 4, Table 5 and Table 6.

## 6. Discussion

The most important finding of the present study is the comparable outcomes between the two cohorts. The telerehabilitation program was effective after TKA and yielded clinical outcomes that were not inferior to conventional outpatient protocols. Looking at the provided metrics and observed differences in outcomes between the considered groups, none of them reported consistently better results, also when looking at the small effect sizes. This may lead to confirming the proposed hypothesis of non-inferiority of telerehabilitation post-operative treatment in TKA with respect to the standard in-presence approach. The primary outcome (physical and balance performance and walking autonomy), measured through the TUG test, let us confirm our preliminary hypothesis about the effectiveness of telerehabilitation in TKA patients (with a median reduction of 8 s from baseline—40% of the initial score). As detailed above all the other investigated clinical parameters showed considerable improvements at 12-week follow-up. This is a further step in the world of telerehabilitation which will significantly develop in the next years and progressively change rehabilitative paradigms.

Telerehabilitation has been developed in the last decades, to allow health professionals to remotely monitor rehabilitation programs with subsequent improved patient adherence to physiotherapeutic protocols [25,26]. Several studies have examined and confirmed the technical feasibility of in-home telerehabilitation [27,28,29]. Although several recent reports and systematic reviews have explored the efficacy of these services [13,26,30,31,32], the successful implementation of telerehabilitation remains slow [33]. This is due to a certain skepticism among healthcare professionals and patients. Despite these difficulties in telemedicine (and telerehabilitation) diffusion, the Italian Ministry of Health in the last 5 years has shown an increasing interest in the argument, so telemedicine guidelines have been developed for redesigning the Italian National Health System [34]. Furthermore, on the global stage, the COVID-19 pandemic led the World Confederation for Physical Therapy’s task force to decisively promote telehealth physical therapy and service paradigms within medical centers to limit the collateral damage to the users of rehabilitation as much as possible [35].

Even in our therapeutic context (due to the COVID-19 outbreak) it was necessary to modify in a short time the traditional physiotherapeutic approach for patients undergoing TKA. We hypothesized that a home-based telerehabilitation program (through the remote supervision performed by physiotherapists) could grant an adequate post-operative recovery, with clinical outcomes comparable to conventional in-presence care. The use of telerehabilitation may improve patient compliance (especially of employed patients), facilitate participation (especially when patients are not able to travel or reach rehabilitation centers due to geographic challenges in structurally weak areas or when appropriate healthcare services are missing), and reduce costs [36] and time [27]. Telerehabilitation can assist homebound patients without the physical presence of health professionals [37,38] but involves many challenges, such as technical reliability, costs of internet/communication technologies, and user-friendliness of the equipment. The increasing availability of user-friendly apps and low-cost internet services has boosted the opportunity to provide telerehabilitation [39]. The majority of the research in post-surgical orthopedic rehabilitation has focused on TKA showing that telerehabilitation is effective [27,40,41,42,43]. The results of our study confirm our preliminary hypothesis and are consistent with those obtained by Shim et al. who examined 56 patients with TKA participating in digital healthcare rehabilitation or conventional rehabilitation group. The authors measured and recorded 4-m gait speed, health-related quality of life, and daily activities [assessed by the EuroQoL 5-Dimension 5-Level (EQ5D5L) questionnaire and by the Western Ontario and McMaster Universities Osteoarthritis Index (WOMAC) score], pain [measured using a numeric rating scale (NRS)], Berg Balance Scale (BBS), range of motion (ROM), and muscle strength with a 24-week follow-up. No group difference in 4-m gait speed and no significant group-by-time interaction regarding 4-m gait speed, NRS, EQ5D5L, WOMAC, BBS, ROM, and muscle strength score was observed [44].

All participants of our study agreed that telerehabilitation was a good alternative to in-person physiotherapy, going beyond the limits of social distancing, imposed by COVID-19 pandemic management. In addition, the elimination of all transportation time and decreased preparation time to get ready for the physiotherapeutic appointment were additional advantages. Though the physical distance imposed by telerehabilitation, all participants developed a good relationship with their therapist, perceived as an important support to improve their physical and mental condition, as if the physiotherapist was there in person. We are aware that a strong patient-therapist relationship should be preserved despite the virtual nature of the interaction. Getting rid of the initial concern due to the lack of therapist in-person contact, patients perceived that the therapists provided appropriate supervision anyway and were able to define a tailored rehabilitation program, according to their ability. All participants found the adopted technology easy to use, with little initial inconvenience, solved by caregivers or therapists.

These issues are consistent with those of a systematic review performed by Brigo et al. which examined the role of telehealth to guarantee the continuity of rehabilitation during the COVID-19 pandemic. The review included 20 studies for a total of 224,806 subjects (93.1% with orthopedic complaints and 6.9% with non-orthopedic ones) and confirmed that telerehabilitation was a safe option to remotely deliver rehabilitation information while respecting social distancing, reducing the risk of infection and the burden of travel [45]. There is increasing evidence in Literature to support orthopedic telerehabilitation, due to its positive effects on various clinical conditions (including TKA), though the investigated telerehabilitation systems differ in terms of implemented features [46,47,48,49]. In the last years, we have experimented the need for implementing telehealth services in total joint replacement orthopedic practice due to the higher age of the patients and the risks of complications from COVID-19 [50]. A systematic review performed by Petersen et al. in 2021 (with a literature search examining randomized controlled trials on telemedical applications in orthopedics) found no difference between telerehabilitation and conventional rehabilitation after joint arthroplasty of the lower extremity regarding functional outcome parameters and PROMs [51]. Even Internet-based rehabilitation (as investigated by Wang et al. in 2023 by including in their review eleven studies with 1020 participants) has comparable effectiveness to face-to-face rehabilitation on rehabilitation outcomes among patients after total joint arthroplasty. No significant difference in outcomes of pain (SMD −0.11, 95% CI −0.32 to 0.10), range of motion in flexion (MD 0.65, 95% CI −1.18 to 2.48), and extension (MD −0.38, 95% CI −1.16 to 0.40), patient-reported physical function (SMD 0.01, 95% CI −0.15 to 0.17), health-related quality of life (SMD −0.09, 95% CI −0.26 to 0.07), satisfaction (SMD −0.04, 95% CI −0.21 to 0.14), and psychological well-being (SMD 0.10, 95% CI −0.13 to 0.33) have been found. Moreover, better outcomes in physical functional tests were obtained by patients performing Internet-based telerehabilitation (SMD −0.54, 95% CI −1.08 to −0.01) [42].

Therefore telerehabilitation seems to be a promising option for the recovery of motor function after orthopedic surgery, useful to face the challenges of everyday life [42,52,53]. According to preliminary evidence, telerehabilitation program is associated with good clinical outcomes, comparable to traditional rehabilitation. In 2011 Russell et al. demonstrated that telerehabilitation outcomes were comparable to in-person traditional rehabilitation in patients undergoing TKA in terms of muscle strength, range of motion, pain, and quality of life [40]. These findings were confirmed by Tousignant et al. who showed home telerehabilitation efficacy in improving function (walking, knee function, and autonomy) and reducing disability (muscle strength, range of motion, and balance) after two months of physiotherapeutic sessions [27]. In the same way, an integrative review performed by Wang et al. (including 22 eligible studies with 1179 subjects) reported that internet-based telerehabilitation provided comparable improvements in terms of pain reduction, improvement in articular range of motion, physical function, and quality of life when compared to in-presence rehabilitation [42]. Similarly, Piqueras et al. demonstrated that a two-week interactive telerehabilitation is as effective as traditional in-presence therapy [54]. Kalron et al. showed that a physical therapy program (3 sessions/week) associated with a telerehabilitation program (video clips of common exercises; 6 weeks) was more effective than conventional physiotherapy [55]. This last piece of evidence was confirmed by Dias Correia et al. who showed better outcomes after an-8 week telerehabilitation program compared to traditional physiotherapy [56]. Agostini et al. in their meta-analysis performed in 2015 showed measurably superior clinical results for patients treated with telerehabilitation following total knee replacement surgery [53]. Similarly Seron et al., despite the contradictory results of their review, highlighted that telerehabilitation in physical therapy could be comparable with in-person rehabilitation or better than no rehabilitation for different orthopedic conditions such as low-back pain, osteoarthritis, and knee and hip arthroplasty [11]. These conclusions were recently indirectly confirmed by LeBrun et al. (in 2022) who performed a retrospective matched cohort study of 326 TKA patients, comparing the safety and efficacy of an institutional telerehabilitation program with those of a conventional “face-to-face” rehabilitation. The authors recorded similar patient-reported outcomes in the Knee Injury and Osteoarthritis Outcome Score for Joint Replacement (KOOS-JR), pain VAS, Veterans RAND 12 (VR-12), and Lower-Extremity Activity Scale (LEAS), thus suggesting that telerehabilitation could grant an equally effective alternative to conventional post-operative rehabilitation following total knee replacement [57]. These conclusions were more recently similarly signed by Summers et al. who compared the clinical outcomes of 135 consecutive TKA patients receiving a home-based clinician-controlled therapy system and those of 135 consecutive patients receiving standard therapy protocol at 2, 6, and 12 weeks, based on pain VAS, ROM, and KOOS JR results. Pain VAS and KOOS JR outcomes were statistically better (*p* < 0.001) and exceeded the threshold for the minimal clinically important difference in the telerehabilitation group. In the same way, knee ROM was greater in the telerehabilitation cohort and was associated with a low risk of arthrofibrosis requiring manipulation under anesthesia. On these bases, the author concluded that this innovative telerehabilitation modality was superior to standard therapy protocol [58].

The results of our study confirm that not only the patient’s clinical outcome but also the levels of satisfaction after using telerehabilitation for TKA were comparable to face-to-face interventions [27,38,40,43,44,59,60]. Our satisfaction results reflect what emerged from another study performed by Negrini et al. during the Italian COVID-19 lockdown, where a high level of satisfaction (2.8 out of 3) derived from the use of telemedicine, provided to patients with spinal disorders [61]. Similarly, in a study published by Nuevo et al. in 2023 and examining telerehabilitation following TKA, patients expressed high satisfaction indicating that they would recommend this therapeutic modality and would like to use it again in the future [62]. Though the concept of satisfaction is complex, being related to various aspects (including the overall organization of care and the relationship between the patient and the therapist) [63], it has often been used as one of the indicators of quality in healthcare, due to its ability to influence adherence to treatment plan and enhance clinical outcomes [64]. High satisfaction rates are determined by three predetermined factors: the health care services, the technology, and the relationship with the health care professional using validated questionnaires. Patients’ perceptions can have a significant impact on rehabilitation outcomes but few studies have explored the patient’s perspective regarding telerehabilitation [38]. As confirmed by Berton et al. in their meta-analysis age and social backgrounds can influence treatment adherence, but the relationship between patient and therapist is equally important. The authors highlight the fundamental role of telehealth technologies even in terms of cost, given the growing demand for orthopedic treatment and the associated rising costs [60]. On the same line, Nelson et al. in 2021 concluded in their trial-based economic evaluation that telerehabilitation (considering even the treatment adherence) is more cost-effective and efficient than face-to-face rehabilitation care for total hip arthroplasty patients [36]. Tousignant et al. in their study published in 2015 came to the same conclusions, reporting that telerehabilitation was more cost-effective as far as the distance between patient and therapist is >30 km [65].

Although the notable findings, there are several limitations in this pilot study. First of all, due to the pandemic emergency, we had to re-modulate our rehabilitation strategies in a short time, to guarantee an adequate and safe post-operative path, aware of the importance of timing for better functional outcomes after total knee replacement. These preconditions led us to use a ready-for-use easy tool, though there are more elaborate platforms, tested and described in previous studies [65,66]. Second, this pilot study (with a relatively small number of patients) was a retrospective analysis and some baseline scores differences in clinical measures between the two groups were observed. Anyway, dishomogeneity was not consistent in a single direction: telerehabilitation group reported better performances in ROM, while in-presence rehabilitation group reported better baseline condition in pain and, in both cases, differences may be considered as clinically neglectable (4° mean difference in flexion and 0.5 points on VAS scale). Third, the psychological impact caused by the COVID-19 pandemic can have conditioned overall outcomes and patients’ satisfaction even though it was hard to precisely define this influence (positive or negative overall effect?). Fourth, the absence of specific instrumental investigations (ultrasonography, electromyography) may limit the quality of the outcomes. Other limitations are represented by the length of the follow-up and by the short physiotherapeutic intervention duration (12 weeks). Therefore we could not determine whether the telerehabilitation program may produce long-lasting benefits in physical function.

## 7. Conclusions

Telerehabilitation is an option for equitable access to rehabilitation services, limiting time, costs, and unnecessary hospital admissions or delays in discharging patients at home. Although in-presence rehabilitation programs still represent the golden standard for the constant physical interaction between patient and physician, telerehabilitation is a reliable alternative in those situations where access to an in-person program is difficult. This change of perspective needs a cultural adaptation not only by patients but first of all by surgeons, physical therapists, entrepreneurs, and rulers. However, further studies are required to determine the optimal blend of telerehabilitation and the traditional in-presence approach.

## Figures and Tables

**Table 1:** Demographic data of the 2 cohorts of patients (telerehab group and in-presence rehabilitation group).

	Telerehab	In-Presence	*p*-Value
**Age**	66.8 ± 10.2	65.4 ± 11.8	0.18
**BMI**	26.8 ± 2.6	27.2 ± 2.9	0.09
**Sex**			0.87
Male	34 (41%)	36 (44%)	
Female	48 (59%)	46 (56%)	
**Side affected**			0.41
Right	24 (30%)	30 (36.6%)	
Left	58 (70%)	52 (63.4%)	
**Education (no. of years completed)**	15.8 ± 3.7	15.0 ± 4.2	0.01
**Work status**			0.58
Employed full or part-time	27%	22%	
Not working, unemployed, unable to work, or retired	73%	78%	
**Comfort with the use of** **technology (tablet, smartphone, or computer)**			0.51
Very comfortable	21.90%	19.50%	
Somewhat comfortable	67.10%	61%	
Somewhat uncomfortable	4.90%	8.50%	
Very uncomfortable	6.10%	11%	

**Table 2:** Clinical measures baseline.

	Telerehab	In-Presence	*p*-Value
**TUG**	20 ± 2	18 ± 1.5	<0.01
**Flexion**	70 ± 3.1	66 ± 8.5	<0.01
**Extension deficit**	8 ± 2	10 ± 1.4	<0.01
**Resting pain**	2.2 ± 0.1	1.7 ± 0.4	<0.01
**Movement pain**	3.6 ± 0.5	3.2 ± 0.4	<0.01
**OKS**	22 ± 1.3	23 ± 2.1	<0.01
**KOOS**	46.2 ± 10.2	48.4 ± 8.4	0.1

**Table 3:** VAS (resting pain and movement pain) evolution between the beginning and end of the physiotherapeutic program (12 weeks).

	TELEREHAB	IN-PRESENCE	Inter-Group Difference *p*-Value	Effect Size
**RESTING PAIN**				
Baseline	2.2 ± 0.1	1.7 ± 0.4		
12 weeks	0.8 ± 0.2	0.6 ± 0.1		
Δ 12 weeks—Baseline	−1.4 ± 0.2	−1.1 ± 0.5	<0.01	0.27
Intra-group changes *p*-value	<0.01	<0.01		
**MOVEMENT PAIN**				
Baseline	3.6 ± 0.5	3.2 ± 0.4		
12 weeks	1.6 ± 0.3	1.2 ± 0.3		
Δ 12 weeks—Baseline	−2 ± 0.6	−2 ± 0.6	0.93	<0.01
Intra-group changes *p*-value	<0.01	<0.01		

**Table 4:** Knee range of motion—ROM—(flexion and extension deficit) evolution between the beginning and end of the physiotherapeutic program (12 weeks).

	TELEREHAB	IN-PRESENCE	Inter-Group Difference *p*-Value	Effect Size
**FLEXION**				
Baseline	70 ± 3.1	66 ± 8.5		
12 weeks	115 ± 5.6	112.1 ± 6.3		
Δ 12 weeks—Baseline	45 ± 6.7	46.1 ± 10.8	0.32	0.08
Intra-group changes *p*-value	<0.01	<0.01		
**EXTENSION DEFICIT**				
Baseline	8 ± 2	10 ± 1.4		
12 weeks	4.1 ± 1.5	5 ± 1.9		
Δ 12 weeks—Baseline	−3.9 ± 2.5	−5 ± 2.3	<0.01	0.23
Intra-group changes *p*-value	<0.01	<0.01		

**Table 5:** Time Up and Go (TUG) test, Oxford Knee Score (OKS) and Knee injury and Osteoarthritis Outcome Score (KOOS) evolutions between the beginning and end of the physiotherapeutic program (12 weeks).

	TELEREHAB	IN-PRESENCE	Inter-Group Difference *p*-Value	Effect Size
**TUG test**				
Baseline	20 ± 2	18 ± 1.5		
12 weeks	12 ± 1.5	13.1 ± 2		
Δ 12 weeks—Baseline	−8 ± 2.6	−4.9 ± 2.5	<0.01	0.5
Intra-group changes *p*-value	<0.01	<0.01		
**OKS**				
Baseline	22 ± 1.3	23 ± 2.1		
12 weeks	36 ± 2.7	35.1 ± 4.2		
Δ 12 weeks—Baseline	14 ± 3.1	12 ± 4.9	<0.01	0.22
Intra-group changes *p*-value	<0.01	<0.01		
**KOOS**				
Baseline	46.2 ± 10.2	48.4 ± 8.4		
12 weeks	67.4 ± 3.8	68.3 ± 6.6		
Δ 12 weeks—Baseline	21.2 ± 11.2	19.8 ± 10.6	0.38	0.07
Intra-group changes *p*-value	<0.01	<0.01		

**Table 6:** Self-administered patient satisfaction scale (Mahomed N, 2011)—Percentage distribution for responses for each item at the end of the physiotherapeutic program (12 weeks).

	TELEREHAB	IN-PRESENCE	*p*-Value
**How satisfied are you with the results of your surgery?**			
Very satisfied	67.1%	68.2%	0.86
Somewhat satisfied	21.9%	24.4%	
Somewhat dissatisfied	6.1%	3.7%	
Very dissatisfied	4.9%	3.7%	
**How satisfied are you with the results of your surgery for improving your pain?**			
Very satisfied	65.8%	63.4%	0.92
Somewhat satisfied	20.7%	21.9%	
Somewhat dissatisfied	7.4%	6.1%	
Very dissatisfied	6.1%	8.6%	
**How satisfied are you with the results of surgery for improving your ability to do home or yard work?**			
Very satisfied	39.0%	43.9%	0.46
Somewhat satisfied	43.9%	46.3%	
Somewhat dissatisfied	9.7%	7.4%	
Very dissatisfied	7.4%	2.4%	
**How satisfied are you with the results of surgery for improving your ability to do recreational activities?**			
Very satisfied	31.7%	34.2%	0.9
Somewhat satisfied	43.9%	46.4	
Somewhat dissatisfied	10.9%	8.5%	
Very dissatisfied	13.5%	10.9%	

## Data Availability

Data are available upon request from the corresponding author.

## Appendix A. Knee Arthroplasty Rehabilitation Protocol: 12-Week Step-by-Step Guide (Instructions for the Patients)

**Week 1–2:**

1. **Pain Management**: Use prescribed pain medications as needed to manage postoperative pain.
2. **Cryotherapy**: Apply ice packs to the surgical area for 15–20 min several times a day to reduce swelling.
3. **Weight-Bearing**: Start with partial weight-bearing as advised by your surgeon and gradually progress to full weight-bearing as tolerated.
4. **Range of Motion (ROM) Exercises**: Perform gentle passive and active-assisted knee flexion and extension exercises to regain knee mobility.
5. **Ankle Pumps and Quad Sets**: Practice ankle pumps and quad sets to maintain circulation and prevent muscle atrophy.

**Week 3–6:**

1. **Weight-Bearing Activities**: Gradually increase weight-bearing activities, such as walking with a walker or crutches, under the guidance of your physical therapist.
2. **Strengthening Exercises**: Start with gentle quadriceps, hamstring, and gluteal muscle strengthening exercises to improve knee stability.
3. **Straight Leg Raises**: Begin straight leg raises to further strengthen the quadriceps muscle.
4. **Stationary Bike**: Incorporate stationary biking with low resistance to improve knee flexion and overall cardiovascular endurance.
5. **Balance and Proprioception**: Practice balance exercises to improve stability and proprioception.

**Week 7–10:**

1. **Progressive Weight-Bearing**: Continue to progress with weight-bearing activities and gradually reduce reliance on walking aids.
2. **Advanced Strengthening**: Introduce more challenging strengthening exercises, such as step-ups, leg presses, and lunges.
3. **Mini Squats**: Initiate mini squats to enhance knee function and range of motion.
4. **Gait Training**: Work on normalizing gait patterns and improving walking mechanics.
5. **Plyometric Exercises**: Incorporate low-impact plyometric exercises, like jumping on a mini-trampoline, to improve dynamic knee stability.

**Week 11–12:**

1. **Functional Activities**: Focus on functional exercises that mimic daily activities, such as stairs climbing, squatting, and getting in/out of a chair.
2. **Balance and Coordination**: Engage in more challenging balance and coordination exercises to improve overall lower limb function.
3. **Endurance Training**: Increase the intensity and duration of stationary biking or other low-impact cardiovascular exercises.
4. **Return-to-Activity Preparation**: Gradually integrate activities specific to your daily routines and hobbies.
